# Stitching Locally Fitted T-Splines for Fast Fitting of Large-Scale Freeform Point Clouds

**DOI:** 10.3390/s23249816

**Published:** 2023-12-14

**Authors:** Jian Wang, Sheng Bi, Wenkang Liu, Liping Zhou, Tukun Li, Iain Macleod, Richard Leach

**Affiliations:** 1 State Key Laboratory of Digital Manufacturing Equipment and Technology, Huazhong University of Science and Technology, Wuhan 430074, China; jianwang@hust.edu.cn (J.W.); bishengjiran@hust.edu.cn (S.B.); m202170450@hust.edu.cn (W.L.); 2 Centre for Precision Technologies, University of Huddersfield, Huddersfield HD1 3DH, UK; t.li@hud.ac.uk; 3 IMA Ltd., 29 Clay Lane, Hale, Cheshire WA15 8PJ, UK; 4 Faculty of Engineering, University of Nottingham, Nottingham NG8 1BB, UK

**Keywords:** T-spline, freeform fitting, large-scale point cloud, stitching splines

## Abstract

Parametric splines are popular tools for precision optical metrology of complex freeform surfaces. However, as a promising topologically unconstrained solution, existing T-spline fitting techniques, such as improved global fitting, local fitting, and split-connect algorithms, still suffer the problems of low computational efficiency, especially in the case of large data scales and high accuracy requirements. This paper proposes a speed-improved algorithm for fast, large-scale freeform point cloud fitting by stitching locally fitted T-splines through three steps of localized operations. Experiments show that the proposed algorithm produces a three-to-eightfold efficiency improvement from the global and local fitting algorithms, and a two-to-fourfold improvement from the latest split-connect algorithm, in high-accuracy and large-scale fitting scenarios. A classical Lena image study showed that the algorithm is at least twice as fast as the split-connect algorithm using fewer than 80% control points of the latter.

## 1. Introduction

In precision optical metrology, point cloud data processing enables high-precision shape measurement and surface reconstruction, which is important for applications requiring very high measurement accuracy, such as engineering quality control, product inspection, and reverse engineering [1,2,3]. In the realm of smart sensing, point cloud data processing finds critical utility in environmental sensing and obstacle detection, addressing applications of paramount importance [4,5,6]. Consequently, point cloud data processing plays a crucial role in both smart sensing and precision optical metrology, offering essential tools and a data foundation for resolving intricate challenges. Point cloud data consist of a large number of three-dimensional (3D) coordinate points, which are usually obtained by optical measurement devices, such as light detection and ranging (LIDAR) and 3D scanning [7]. At present, advanced optical scanning has enabled the acquisition of over ten million 3D point clouds within a second [8,9]. For precision metrology of surfaces with complex freeform geometry, dense point cloud acquisition has become an ordinary measurement routine, using which a denoised point cloud, polygon mesh, or CAD model is finally fitted [10]. The spline technique has clearly become an indispensable part of surface fitting in point cloud data processing due to its many advantages such as smoothness, mathematical feasibility, and controllability [11].

Splines are widely used in interpolation, smoothing, and modeling in computer-aided geometric design, metrology, graphics, and digital signal processing [12]. With the development of powerful node insertion and order elevation algorithms, B-splines and non-uniform rational B-splines (NURBS) have become the mainstream mathematical methods of shape description [13]. B-splines and NURBS are also CAD models in line with industrial standards. Due to the limitation in topology, however, a NURBS surface model may have many redundant control points (CPs), seriously hindering the design of complex geometries. Many improved B-spline variants, such as truncated hierarchical B-splines and locally refined T-splines, which are well known for their adequate local controllability [14,15], have been developed with limited CPs. Among them, Sederberg [16,17] T-splines which allow T-junctions in control meshes could be the most promising options, and as a result of their commercial success, they have been integrated into Autodesk Fusion360. T-splines have received rapid development, given their excellent topological characteristics. Hierarchical T-splines, analysis-suitable T-splines (AST-splines), truncated T-splines, and various modified T-splines [18,19,20,21,22,23] have been developed from there. Recently, they have also been successfully applied to iso-geometrical analyses [23,24]. For these large-scale complex geometry data, existing T-spline fitting algorithms suffer severe efficiency problems compared to NURBS, especially when high fitting accuracy is required. To address this problem, the existing T-spline fitting algorithms must be analyzed regarding their strategic weaknesses.

Existing T-spline fitting algorithms usually follow a top-down or bottom-up iterative computational strategy. Zheng [25] developed the first global T-spline fitting algorithm for simple z-map data in the form of z=f(x,y). They mentioned that the calculation is very time-consuming when the number of data points is large. Wang [26] considered using surface curvature to guide T-mesh refinement, so more CPs are automatically allocated in regions with rich features. This feature-guided strategy improves computational efficiency to a certain extent. Global AST-spline fitting algorithms have also been developed using the spatial distribution of sampling points, feature points, and fitting error points for local refinement guidance [27]. T-splines allowing local refinement are welcome for their topologically unconstrained parametric meshes. However, their basis functions have increased computational complexity over B-splines and may not share a partition of unity, and as a result, global T-spline fitting is usually slower than topologically simple splines, e.g., multi-level and truncated hierarchical B-splines [28]. The efficiency problem is especially critical if a T-spline model is topologically more restrictive, such as AST-splines [19].

Following the global fitting framework, fast T-spline fitting techniques have been investigated. Chen [29] developed a Bézier extraction framework for hierarchical T-splines such that explicit basis function calculations are avoided, leaving matrix manipulations to be the only computational task. Wang [30] proposed an efficient data structure to store the topology information of unstructured T-splines, with which an effective local parameterization algorithm was developed to improve the fitting efficiency. Lin and Zhang [31] presented a progressive T-spline fitting algorithm for large data sets based on preconditioned conjugate gradient optimization. The iteration speed of the method is steady and insensitive to the growing number of unknown T-mesh points. Kermarrec [32] proposed a local surface approximation algorithm called multilevel T-splines approximation, which can stably deal with large and noisy point clouds with variable point densities.

Recently, Feng and Taguchi [33] proposed a completely different, bottom-up framework, known as “split-connect”, for fast T-spline fitting. This method shows an order of magnitude faster than classical global methods. However, it neglects the local support of T-spline basis functions and the support influence of CPs on surrounding patches, resulting in the fitting accuracy being hard to control and with redundant CPs. Since 2020, Wang [34] and Lu [35] separately developed locally accelerated algorithms for T-spline fitting, or simply local T-spline fitting. They show that a time cost reduction of two thirds from the global methods has been achieved. Their uniqueness is that a local instead of global CP fit is used during each spline update iteration. Due to the local characteristics, the efficiency improvement may vanish if a fitted surface does not possess a sparse property [34].

In this paper, we propose a locally accelerated stitching T-spline (LAST) algorithm for large-scale freeform point cloud fitting. LAST breaks through the efficiency bottleneck of data scales by dividing a sizable fitting problem into several minor local fitting problems. LAST does not follow a top-down nor a bottom-up strategy but adopts both. LAST consists of three computational steps with three localized operations. Firstly, LAST divides a large-scale input point cloud into several local patches. Then each data patch is independently fitted to a T-spline through a locally accelerated fitting scheme. Lastly, the individually obtained T-splines are stitched using a local optimization scheme to a global output to ensure their mutual continuities. LAST results in concise T-mesh structures with a dramatic CP-saving performance by preserving the local support characteristics of CPs. We have compared the performance of LAST against the latest techniques, including a global fitting algorithm, a local fitting algorithm, and a split-connect algorithm. The results show that the computational speed is about two-to-eightfold faster than the compared algorithms. In the classical Lena RGB image case study, LAST is shown to be at least twice as fast as the split-connect algorithm, using fewer than 80% CPs of the latter. The proposed method can also be applied with advanced T-spline models, such as AST-splines, and hierarchical T-splines [18,19,20,21,22,23], providing a corresponding mesh refinement rule.

## 2. State-of-the-Art Algorithm Analyses

### 2.1. T-Spline Fundamentals

**Figure 1:**
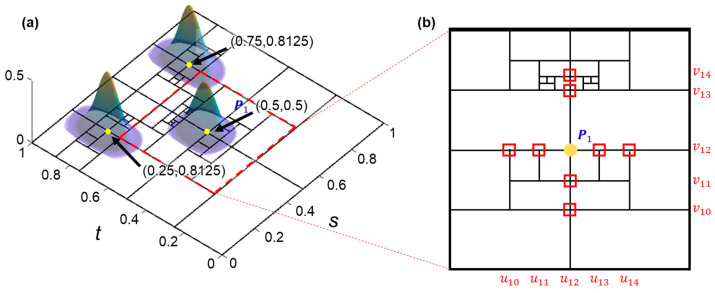
The principle of T-splines. (**a**) T-spline blending functions; (**b**) the T-mesh and the knots (red squares) of the blending function for control point $P_{1}$ corresponding to (**a**).

A 3D T-spline $\bar{q}(s,t)\in R^{3}$ defined on a control mesh of n-CPs allowing T-junctions, known for the T-mesh as presented in Figure 1a, is similar to a NURBS in the form of

(1) $$\bar{q}\left(s,t\right)=\frac{\sum_{i=1}^{n}p_{i}b_{i}\left(s,t\right)}{\sum_{i=1}^{n}b_{i}\left(s,t\right)}=\sum_{i=1}^{n}p_{i}\frac{b_{i}\left(s,t\right)}{\sum_{i=1}^{n}b_{i}\left(s,t\right)}={\left[\sum_{i=1}^{n}b_{i}\left(s,t\right)\right]}^{-1}b^{T}P$$

where $(s,t)\in R^{2}$ is a parametric location mapped, e.g., using conformal mapping [36] (or simply normalized x and y coordinates), from a Cartesian measured point (x,y,z) to a parametric space; P is an *n*-row CP matrix, each row of which represents a CP $p_{i}\in R^{3}$; b is a T-spline blending [17] vector with the *i*-th blending function entry $b_{i}\left(s,t\right)$ in the form of:

(2) $$b_{i}\left(s,t\right)=N_{i,k}\left(s\right)\cdot N_{i,k}\left(t\right)=N\left[u_{i}\right]\left(s\right)\cdot N\left[v_{i}\right]\left(t\right)$$

where $N\left[u_{i}\right]\left(s\right)$ and $N\left[\nu_{i}\right]\left(t\right)$ are B-spline basis functions related to knot vectors $u_{i}$ and $\nu_{i}$, respectively. T-spline knot vectors are uniquely determined by the topology of a T-mesh obtained through Sederberg’s T-spline Rule 1 [16], for example in Figure 1b.

As presented in Figure 1a, a T-spline blending function is a locally supported B-spline basis function that can be recursively calculated using de Boor–Cox’s recursion formulae [13]. Given the knot vectors $u_{i}=[u_{i0},u_{i1},u_{i2},u_{i3},u_{i4}]$ and $v_{i}=[v_{i0},v_{i1},v_{i2},v_{i3},v_{i4}]$ of a natural $C^{2}$ bicubic spline with the multiplicity of one, the recursively obtained blending functions have the following forms, with which repetitive recursion calculations can be avoided in practical calculations:

(3) $$N\left[u_{i}\right]\left(s\right)=\left\{\begin{array}{l} \frac{{\left(s-u_{i0}\right)}^{3}}{\left(u_{i1}-u_{i0}\right)\left(u_{i3}-u_{i0}\right)\left(u_{i2}-u_{i0}\right)},\;\mathrm{for}\;s\in \left(u_{i0},u_{i1}\right],\;\\ \begin{array}{c} \begin{array}{l} \frac{{\left(s-u_{i0}\right)}^{2}\left(u_{i2}-s\right)}{\left(u_{i2}-u_{i1}\right)\left(u_{i3}-u_{i0}\right)\left(u_{i2}-u_{i0}\right)}+\frac{\left(u_{i3}-s\right)\left(s-u_{i0}\right)\left(s-u_{i1}\right)}{\left(u_{i2}-u_{i1}\right)\left(u_{i3}-u_{i1}\right)\left(u_{i3}-u_{i0}\right)}+\frac{{\left(s-u_{i1}\right)}^{2}\left(u_{i4}-s\right)}{\left(u_{i2}-u_{i1}\right)\left(u_{i4}-u_{i1}\right)\left(u_{i3}-u_{i1}\right)},\;\\ \;\mathrm{for}\;s\in \left(u_{i1},u_{i2}\right], \end{array}\\ \begin{array}{l} \frac{{\left(u_{i3}-s\right)}^{2}\left(s-u_{i0}\right)}{\left(u_{i3}-u_{i2}\right)\left(u_{i3}-u_{i1}\right)\left(u_{i3}-u_{i0}\right)}+\frac{\left(u_{i4}-s\right)\left(u_{i3}-s\right)\left(s-u_{i1}\right)}{\left(u_{i3}-u_{i2}\right)\left(u_{i4}-u_{i1}\right)\left(u_{i3}-u_{i1}\right)}+\frac{{\left(u_{i4}-s\right)}^{2}\left(s-u_{i2}\right)}{\left(u_{i3}-u_{i2}\right)\left(u_{i4}-u_{i2}\right)\left(u_{i4}-u_{i1}\right)},\;\\ \;\mathrm{for}\;s\in \left(u_{i2},u_{i3}\right], \end{array} \end{array}\\ \frac{{\left(u_{i4}-s\right)}^{3}}{\left(u_{i4}-u_{i3}\right)\left(u_{i4}-u_{i2}\right)\left(u_{i4}-u_{i1}\right)},\;\mathrm{for}\;s\in \left(u_{i3},u_{i4}\right],\\ 0,\;\mathrm{for}\;\mathrm{other}\;s. \end{array}\right.$$

T-splines break the topological limitation of B-splines and realize local refinement. This indicates that CPs can be locally allocated around a feature without inserting CPs in a whole row or column. T-splines are a generalization of NURBS, both of which can convert mutually. Research has shown that the CP amounts reduce to at least one quarter of the original in many computer-aided design cases when a NURBS simplifies to a T-spline, on the premise of a small accuracy difference [17].

### 2.2. Global T-Spline Fitting

**Figure 2:**
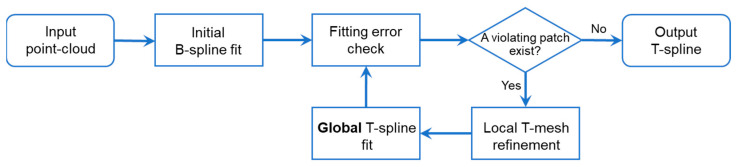
Flowcharts of the global T-spline fitting algorithms.

A top-down global T-spline fitting normally approximates from a regular tensor-product B-spline grid, such as in Figure 2. In most cases, a B-grid is extended around its boundaries using additional CPs for improved boundary geometrical control, including both surface heights and their partial derivatives [34]. The regular grid is then locally refined by inserting CPs and splitting edges at the violating grids or elements where fitting errors exceed a preset threshold in a repetitive way.

**Figure 3:**
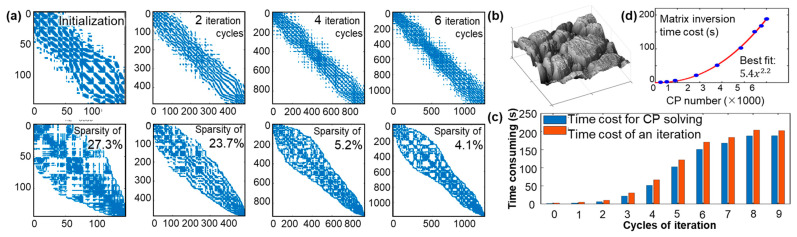
Performance analysis of typical T-spline fitting algorithms for a stainless steel complex surface. (**a**) Sparsity view (top) and corresponding reverse Cuthill–McKee ordering (bottom) of the transposed product of design matrices ${\hat{B}}^{T}\hat{B}$ during each iteration cycle in a global fitting scheme; (**b**) the stainless steel complex surface; (**c**,**d**) time consumption statistics and matrix inversion time costs for a global T-spline fitting calculation.

During each iterative refinement, the CP matrix P updates by solving the following least-squares problem

(4) $$\min\sum_{j=1}^{m}{\left\|\left.q_{j}-\bar{q}\left(s_{j},t_{j}\right)\right\|\right.}^{2}=\min\left\|{\left.Q-\bar{Q}\right\|}^{2}\right.=\min\left\|{\left.Q-\overset{⌢}{B}P\right\|}^{2}\right.=\min\left\|{\left.Q-{\mathrm{diag}}^{-1}\left[\sum_{i=1}^{n}b_{i}\left(s_{j},t_{j}\right)\right]BP\right\|}^{2}\right.$$

where Q is an m-by-3 measured point cloud matrix, each row representing an measured point $q_{i}=\left(x_{i},y_{i},z_{i}\right)$; $B={\left[b\left(s_{1},t_{1}\right),\ldots ,b\left(s_{m},t_{m}\right)\right]}^{T}$ is an *m*-by-*n* T-spline blending matrix consisting of m (m≫n) blending vectors corresponding to each measured point in the direction of columns; $\hat{B}$ denotes the corresponding normalized version of ***B***, known as the T-spline design matrix; ***B*** and $\hat{B}$ are generally sparse, whose sparsity patterns for matrix inversion are presented in Figure 3a, due to the local support of blending functions.

In global strategies, if a violating grid element is refined, the newly inserted CPs and the original CPs are updated from all the input sample data through Equation (4). This global strategy results in tedious calculations, including repetitive design matrix updates and their pseudo-inverse. Figure 3c,d shows an experimental fitting analysis of a stainless steel surface in Figure 3b. It indicates that the spline model complexity quickly increased in the initial iteration cycles. During the last several cycles until the computation converged, the CP number grew slowly and finally approached zero. Simultaneously, the time cost for CP-solving continued to increase, and the design matrix’s sparsity gradually converged to a small percentage. Figure 3c,d also shows that most computing resources focus on the pseudo-inverse-based CP-solving process. The sparsity patterns of the design matrices result in an efficiency-considerable computational complexity in $O(n^{2.2})$ for matrix inversion regarding the number of CPs. In large-scale point cloud and high-accuracy fitting cases, a design matrix may reach over a million rows and a thousand columns, resulting in it being nearly impossible to solve the least-squares problem.

### 2.3. Local T-Spline Fitting

**Figure 4:**
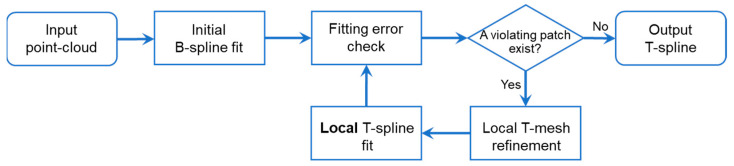
Flowcharts of the local T-spline fitting algorithms.

Locally accelerated T-spline fitting, or simply local T-spline fitting, as an accelerated version of global algorithms, has been proposed in our previous work [27,34]. The local algorithms follow the same top-down strategy as the global algorithms, as shown in Figure 4, including iterative mesh refinement and CP updates. The core difference is that the local algorithms only update the support-altered local CPs around error-violating meshes. In this way, the matrix multiplication $\hat{B}P$ in Equation (1) disassembles into a varying part ${\hat{B}}_{ML}P_{L}$ with the local support-altered CPs to update, plus an unchanged part ${\hat{B}}_{M(N-L)}P_{N-L}$, where M denotes the index set of the measured points participating in the fitting; L and N−L denote, respectively, the index sets of the support-altered and support-unchanged CPs. In AST-spline cases [27] where the partition of unity of blending functions always holds, the matrix partition can be simply written as

(5) $${\bar{Q}}_{M}={\overset{⌢}{B}}_{M\times N}P_{N}={\overset{⌢}{B}}_{M\times L}^{}P_{L}^{}+{\overset{⌢}{B}}_{M\times \left(N-L\right)}^{}P_{N-L}^{}$$

Therefore, by substituting Equation (5) in Equation (4), the local CPs to update may be significantly accelerated by solving the pseudo-inverse of the size-reduced CP matrix $P_{L}^{1}$ only, focusing on the limited areas with complex local geometries. It can also be noted that in AST-spline cases, the unchanged design matrix part ${\hat{B}}_{M(N-L)}$ always stays the same as that of the last iteration, so repetitive design matrix updates are also avoided. With the local acceleration, a large-scale least-squares problem becomes minor and solvable.

**Figure 5:**
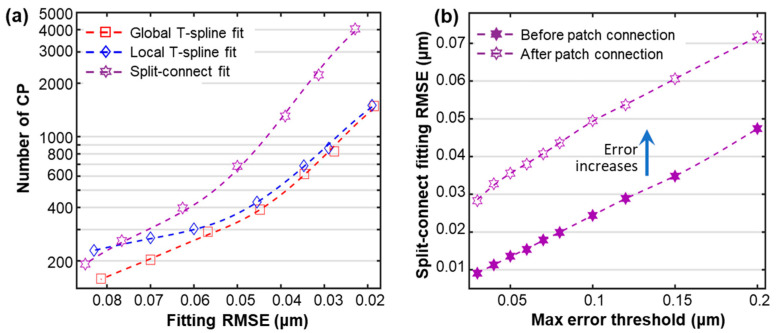
Performance analysis of typical T-spline fitting algorithms for a stainless steel complex surface from Figure 3b. (**a**) Resulting model CP statistics of different algorithms; (**b**) the fitting accuracy degeneration phenomenon in split-connect calculations. Dashed trend lines are fitted using moving averages.

The local computation task may be further accelerated by only using a subset of the input m-point cloud, e.g., by only using the neighbor point sets within the support $P_{L}$. Figure 5a shows that the local fitting method reduces fitting time costs to half that of the global method in relatively high-accuracy cases, coinciding with previous studies. In low-accuracy cases, an initial B-spline fit plus limited refinement iterations may converge, with the result that the efficiency superiority could be not noticeable. In addition, the efficiency improvement may also vanish due to the local computation characteristic if a fitted surface does not possess a sparse property [34], e.g., a surface has a uniform random spatial distribution of features, like hills and valleys. In the latter extreme situation or if the required fitting accuracy is very high, the local fitting algorithm may degenerate into a general global one.

### 2.4. Split-Connect Fitting

**Figure 6:**
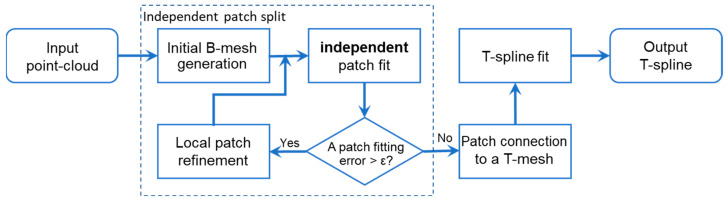
Flowcharts of the split-connect T-spline fitting algorithms.

Split-connect algorithms [33] use a bottom-up approach to fit T-splines, thus avoiding conventional large-scale iteration problems. As presented in Figure 6, a split-connect algorithm includes the following three processes:

1. Independent patch split: Iteratively fit an input point cloud to simple, small, and independent Bézier- or B-spline patches until a pre-set error threshold is achieved. Each spline patch corresponds to a rectangular grid of different sizes in a parameter domain. This step is similar to the refinement iteration of a global or local fitting scheme. The core difference is that the obtained patches here are topologically independent, i.e., the local support of a CP regarding its neighbor patches is neglected, and thus, the fitting of all the separate patches can be very fast.
2. Patch connection: Connect independent spline patches to a T-mesh with a prescribed continuity according to actual data continuity (to avoid missing data problems) and infer the knot vectors of all CPs.
3. T-spline fitting: Calculate the T-spline CPs. Since CPs have been obtained in the first step, though they are inaccurate, optimization of the CPs using a preconditioned conjugate gradient (PCG) algorithm [31] has proven to be remarkably faster than general least squares.

Split-connect has proved to be an order of magnitude faster than classical global algorithms, attributed to the local support ignorance during its initial patch split. However, the final T-spline optimization process reconsiders the local support characteristics of CPs and geometrical continuities between split patches, with the result that initially obtained fitting accuracy cannot hold to the end. An accuracy degeneration phenomenon shown in Figure 5b indicates that the final resulting T-spline’s root-mean-square error (RMSE) is usually twice the value before a final patch connection, and it could be fourfold or even larger than a pre-set maximum fitting error threshold [10]. Therefore, specifying a smaller error threshold is usually required in the initial split fitting to satisfy a final accuracy requirement. However, the support ignorance and the smaller error threshold setting in the initial independent split may easily result in superfluous CPs. Figure 5a indicates that the split-connect fitting may produce over twice the CP amounts of the top-down algorithms in high-accuracy cases.

## 3. LAST Fitting

**Figure 7:**
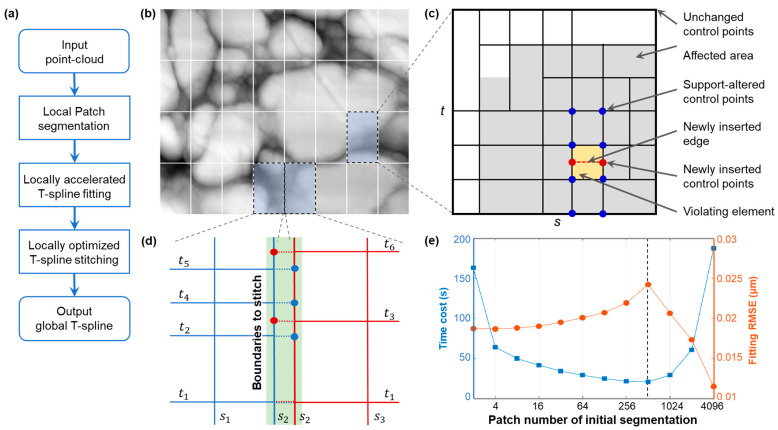
Diagram of LAST fitting algorithm. (**a**) Algorithm flow chart; (**b**) independent patch segmentation of a stainless steel surface topography image for local fit; (**c**) local dimidiate T-spline refinement from a four-CP initial square grid (bold boundaries) and fitting with localized CP topology analysis; (**d**) stitching at the boundaries of two neighbor T-meshes for locally accelerated final T-spline optimization; (**e**) computational time cost and fitting RMSE analysis regarding initially segmented patch numbers for the stainless steel surface. In (**c**), a T-mesh element in yellow is split, and two new CPs in red are inserted. Due to the local support characteristics, only the neighbor (blue) CP-associated blending functions alter. Fitting the local CPs using the local data in the affected grids in grey reduces the complexity of computation significantly; more information is available in the previous work [34].

This section introduces the proposed locally accelerated stitching T-spline fitting algorithm for fast, large-scale freeform point cloud fitting with a high capability of accuracy control. The algorithm combines bottom-up and top-down strategies into a common framework; hence, the advantages of both strategies can be benefited. The algorithm consists of three computational steps of localized acceleration operations, as presented in Figure 7a. Firstly, LAST divides a large-scale input point cloud into several local patches. Then each data patch is independently fitted to a T-spline through a locally accelerated fitting scheme. Lastly, the individually obtained T-splines are stitched using a local optimization scheme to a global output to ensure their mutual continuities. The distinction of LAST from the latest split-connect algorithm is that we segment a point cloud into minor patches for local T-spline fitting, rather than splitting it into totally independent B-spline patches. Also, in the final stitching process, only the local CPs around each patch boundary are locally updated using PCG optimization instead of updating all CPs. The local spline fit-and-stitching behavior results in a balance of computational speed improvement and accuracy maintenance. The algorithm details are explained in the following.

### 3.1. Local Patch Segmentation

Providing an input 3D Cartesian point set to properly map to a parametric space [36], e.g., D=[0,1]⊗[0,1], this step divides the cloud into K minor data patches in the parametric space simply according to sample sizes for subsequent fitting within each patch. Each local patch then comprises an independent T-spline with local-support CPs. Hence, a large-scale fitting problem becomes multiple minor ones. During each local patch fitting, the design matrix dimension in rows reduces to 1/*K* of the original, significantly improving CP-solving speed according to the matrix inversion time curve in Figure 3d.

For a depth map with regular sample points, a uniform segmentation with the same patch size, i.e., the sample size of a patch, is usually expected. Figure 7b shows a four-level hierarchical dimidiate segmentation of a regularly sampled surface depth map. For an irregular point cloud, the segmentation should be adaptive to the distribution of its local point densities. To guarantee that the patches can be finally stitched in a T-spline space, the adaptively segmented patches must be topologically square in a parametric domain. In contrast, the patch sizes can vary, and T-junctions are allowed at their boundaries.

To achieve the highest efficiency, the patch size should be moderate. A too-large patch size results in the LAST fitting deteriorating to a brute-force global fitting; a too-small one may result in the algorithm deteriorating to a bottom-up split-connect fitting with a uniform initial patch split. A numerical analysis regarding the influence of patch segmentation amount on computational time cost and fitting RMSE was carried out, as shown in Figure 7e. It shows that in the early period of patch number increasing, e.g., <512 in this example, the computational time cost reduces until an optimal value is gradually achieved. Simultaneously, the final fitted T-spline RMSE increases gradually. In the larger patch number cases, the time cost grows rapidly, and fitting RMSE significantly decreases simultaneously. This is because the CP number in the latter cases approached the point cloud size, and a quasi-interpolation effect was obtained using a T-spline model. In this study, we used a patch size of 2000 points per patch, e.g., 32 patches in total, as shown in Figure 7a, to conduct the patch segmentation.

### 3.2. Locally Accelerated T-Spline Fitting

Each local point-set patch within a sub-parametric space $D_{k}\in D$ is then individually fitted to a T-spline through a locally accelerated fitting computation. The algorithm is similar to that proposed in previous studies [34,35] but with a few modifications, including mesh initialization and fitting equations.

This process indicates that a T-mesh is iteratively refined in localized areas during a local patch-fitting process, and CP calculation is accelerated by updating only the corresponding local CPs. These local behaviors bring the following benefits: (1) The local refinement behavior of a T-spline ensures control meshes are concise and adaptive to local feature complexities of measured geometry. Thus, limited unknown CPs are introduced during each iteration. (2) The local CP update behavior provides a further dimensional reduction of T-spline design matrix in rows and, thus, a further computational speed boost, according to the partial computation principle as in Equation (5). (3) By involving only the local points for CP update calculation, a further acceleration can be achieved due to a dimensional reduction of the design matrix in rows again.

The local T-spline fitting algorithm consists of the following four minor steps following the flow chart in Figure 4. The last three steps are iteratively applied until all measured points meet a pre-set tolerance.

1. T-spline initialization

As shown in Figure 7c, a simple square grid consisting of four CPs overlaid on F is used as the initial T-mesh of fitting. Boundary extensions [34] are neglected here to ensure patch boundaries are able to be stitched later without topological hindrances. Other tensor-product B-spline grids can also be used in this step for speed optimization. The initial spline can be easily fitted regarding its CPs using Equation (4), by providing the local measured sample points.

2. Tolerance check

To improve fitting accuracy, a T-mesh is iteratively refined to fit an input point cloud with a desired tolerance *σ*. For this purpose, a metric for fitting error evaluation, e.g., max, average, and root-mean-square Euclidean distances between the fitted points $\bar{Q}$ from the measured points Q, are calculated. Then the violating points with a fitting error larger than *σ* and their located mesh patches, e.g., the yellow element in Figure 7c, are identified for further local refinement. If no violating points are found, the iteration stops. In this study, the maximum Euclidean distance from a fitted spline to measured points is used as the threshold metric for the tolerance check.

3. Local refinement

Violating T-mesh patches are split in a dimidiate way for local T-mesh refinement in this step. As presented in Figure 7c, if a rectangle element is split, two associated new CPs are inserted simultaneously. The splitting direction is selected along the longer edge, so a T-mesh retains isotropy during refinement. The dimidiate mechanism and initial rectangle B-spline grids guarantee that all refined patches are always rectangles. To avoid rank-deficiency in later fitting calculations, refinement is only implemented at the patches with more than a specified number (e.g., eight in the study) of data points.

4. Local fitting

A refined T-spline on $D_{k}$ is then locally fitted regarding the newly inserted and support-altered CPs. Along with the insertion of a splitting edge with two new CPs, the blending functions of their surrounding CPs (see Figure 7c, for example) are then altered. This indicates that the blending matrix B, after a local refinement, extends by several new columns, and a limited number of its original columns change. Following the convention in Section 2.3 by indexing the support-alter and newly inserted CPs using L and the remaining CPs using N-L, Equation (5) can be adapted to the general T-splines of Equation (1) in the following form:

(6) $${\bar{Q}}_{M_{-}}={\underset{j\in M_{-}}{\mathrm{diag}}}^{-1}\left[\sum_{i\in N_{-}}b_{i}\left(s_{j},t_{j}\right)\right]\left(B_{M_{-}\times L}^{}P_{L}^{}+B_{M_{-}\times \left(N-L\right)}^{}P_{N-L}^{}\right)$$

where $M_{-}$ denotes an attending subset of measured points, e.g., in the affected elements of Figure 7c, in the accelerated local fitting. The unchanged blending matrix part $B_{M_{-}(N-L)}$ with its associated CPs $P_{N-L}$ can be simply inherited from the last iteration. Thus, if the blending matrix $B_{M_{-}L}$ for the L-indexed CPs and the corresponding summations $\sum_{i}b_{i}(\cdot )$ are calculated, $P_{L}$ can thus be efficiently estimated from the following small-scale least-squares problem:

(7) $$\min{\left\|\underset{j\in M_{-}}{\mathrm{diag}}\left[\sum_{i\in N_{-}}b_{i}\left(s_{j},t_{j}\right)\right]Q_{M_{-}}-B_{M_{-}\times L}^{}P_{L}^{}-B_{M_{-}\times \left(N-L\right)}^{}P_{N-L}^{}\right\|}^{2}$$

### 3.3. Locally Optimized T-Spline Stitching

A global T-spline is obtained finally in the step by stitching all the individual patch T-splines. The global T-spline stitching includes a T-mesh stitching process and a subsequent global T-spline fitting. The T-mesh stitching is carried out by sharing the knots at the overlapping boundaries of two neighbor T-meshes. For example, as presented in Figure 7d, we assume that $s_{2}$ is a parametric position where a left T-mesh and a right one will stitch. The knot vector of the left mesh at $s_{2}$ is $\left[\cdots ,t_{1},t_{2},t_{4},t_{5},\cdots \right]$, while the knot vector of another is $\left[\cdots ,t_{1},t_{3},t_{6},\cdots \right]$. Then, the stitched T-mesh knot vector at the shared boundary on $s_{2}$ becomes $\left[\cdots ,t_{1},{t_{2},t}_{3},t_{4},{t_{5},t}_{6},\cdots \right]$, where repeating knots are eliminated. This boundary CP set union operation ensures the topological conciseness of stitching. Nevertheless, it results in an accuracy loss of finally stitched T-splines due to a reduced total CP number [33]. Extending a boundary CP to the inner edges of a neighboring T-mesh may mitigate the problem.

The set union operation also alters the knot vectors and the supports of boundary CPs. Re-fitting the CPs at the stitching boundaries and their nearby areas is thus needed to guarantee the resulted T-spline accuracy. For this problem, the locally accelerated fitting scheme described in Figure 7c and Equation (7) is used again for a speed concern, instead of updating all spline CPs crassly. However, it should be noted that since the boundary CPs have already been estimated, the local CP-solving problem here is further accelerated using PCG optimization [31] instead of using least squares, as in Section 3.2. Since the stitching process involves CP optimization around patch boundaries, a too-large initial patch segmentation should not be expected as it produces excessive CPs around patch boundaries. Figure 7e shows that the computational time cost in the extreme cases significantly increases because nearly all the CPs are close to patch boundaries and need to be re-fitted.

## 4. Experiments

The LAST fitting algorithm was tested regarding accuracy requirements and data scales, in comparison with a global, a local, and a split-connect fitting algorithm. The experiments were carried out on a Lenovo computer (Intel i7, 2.8 GHz CPU, and 32 GB memory) under a MATLAB 2020a environment without parallel acceleration. Typical surfaces, including a random stainless steel surface, periodically laser-patterned hard-disk surface [32], and classical RGB portrait of Lena, were analyzed. The former two are used to uncover the performance of LAST thoroughly. The Lena image is used as a benchmark for performance comparison against the state-of-the-art fast split-connect algorithm.

### 4.1. High-Accuracy Fitting

**Figure 8:**
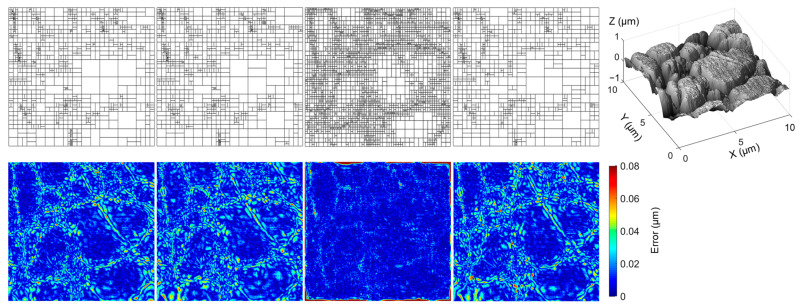
Fitted T-meshes (**top**) with an RMSE of ~0.02 μm and corresponding fitting error maps (**bottom**) for a stainless steel surface. Each column from left to right denotes the global, local, split-connect, LAST fitting result, and the 256 × 256-pixel input map, respectively.

A fitting accuracy analysis of the stainless steel surface was carried out. Typical fitted T-meshes and corresponding fitting error maps of the analyzed algorithms with an equivalent fitting error are presented in Figure 8. It shows that the LAST fitting algorithm produced a T-spline and an error map similar to the global and local algorithms without generating excessive CPs around the patch boundaries. The complex T-spline model obtained from the split-connect algorithm has minor fitting errors in the central area of the parametric space but significant errors at the space boundaries. Conversely, LAST exhibits larger errors in regions with high gradients. All the fitted T-meshes have dense CP distributions around the geometrical features.

**Figure 9:**
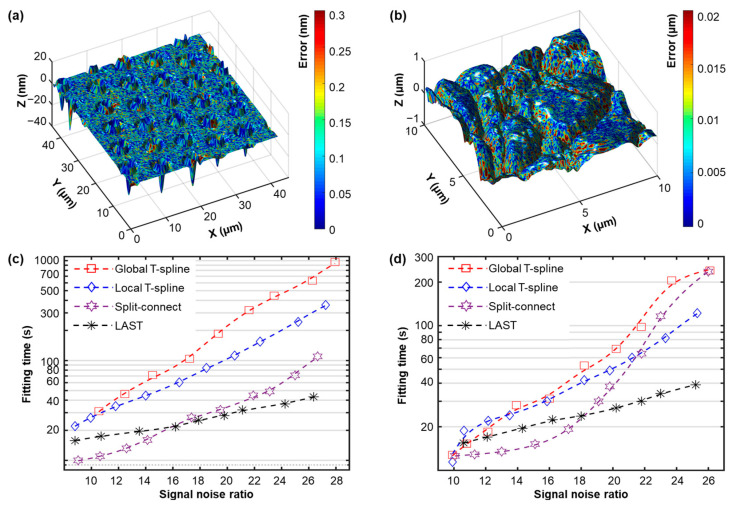
Two typical fitted freeform fitting results using LAST (**a**,**b**) and their computational time cost statistics against competing algorithms at different accuracies (**c**,**d**), for a 170 × 185-pixel laser-patterned surface (**a**,**c**) and a 256 × 256-pixel stainless steel surface (**b**,**d**), respectively. Dashed trend lines are fitted using moving averages.

The efficiency performance in terms of fitting accuracy indexed by the signal-noise ratio of the test algorithms was then analyzed

(8) $$\mathrm{SNR}=10\cdot \log_{10}\frac{\sum_{x=1}^{N_{x}}\sum_{y=1}^{Ny}q{\left(x,y\right)}^{2}}{\sum_{x=1}^{N_{x}}\sum_{y=1}^{Ny}{\left[q\left(x,y\right)-\bar{q}\left(x,y\right)\right]}^{2}}$$

where the scalers $q\left(x,\;y\right),\bar{q}\left(x,y\right)\in R$ describing the heights of a z-map surface are one-dimensional versions of q and $\bar{q}$ in Equations (1)–(4). Figure 9a,b show the typical fitting results of the laser-patterned surface and stainless steel surface. The adaptively fitted T-splines present relatively uniform Euclidian distance point-to-point errors across the surfaces. Figure 9c,d indicate that for both the periodically patterned and randomly patterned surfaces, LAST fitting achieves excellent efficiency performance against the state-of-the-art fitting algorithms. Specifically, in the low-accuracy fitting scenarios, LAST performs faster than the global and local algorithms but slightly worse than the split-connect algorithm. When the fitting accuracy requirement becomes higher, the split-connect algorithm gradually becomes inefficient because the final PCG optimization is time-consuming in large-scale cases. On the contrary, the LAST fitting gradually becomes the fastest algorithm in high-accuracy cases, thanks to the local support reservation in the fitting calculations.

### 4.2. Large-Scale Fitting

**Figure 10:**
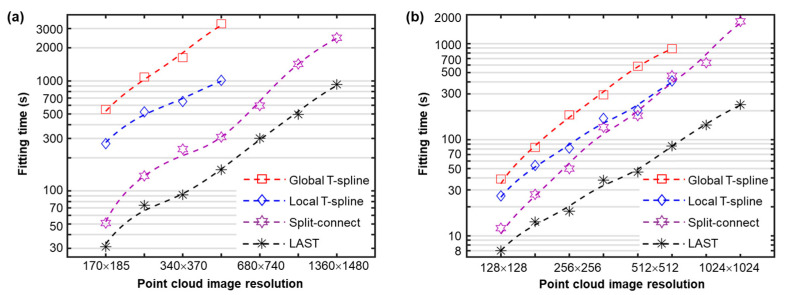
Fitting efficiency analysis against point cloud scales for the laser-patterned surface (**a**) and stainless steel surface (**b**) with an RMSE of 0.21 nm and 0.02 μm, respectively. Dashed trend lines are fitted using moving averages.

Fitting efficiency against point cloud scales was then analyzed. The periodical and random pattern surfaces of different sample sizes were fitted against different accuracy levels, and the corresponding computational time costs were also analyzed, as presented in Figure 10. The approximately linear log-log plots in Figure 10 indicate that the time consumption of the four test algorithms increases in a power function against input data sizes. The LAST fitting has the fewest time elapses consistently among the test algorithms. Specifically, LAST is about two-to-fourfold faster than the existing fast split-connect and local fitting algorithms. The global and local fitting algorithms stopped when the sample size increased to a certain level due to oversized matrix-inversion calculations.

### 4.3. Lena Image Comparison

**Figure 11:**
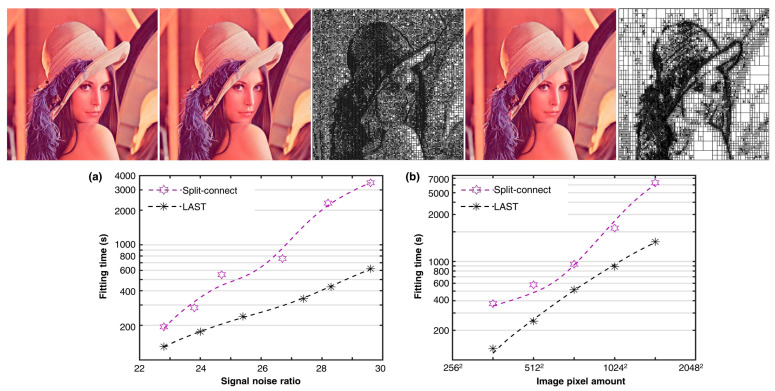
Lena image fitting analysis. Top: Each column from left to right represents the input image, the fitted image, and corresponding 32,140-CP T-mesh of the split-connect fitting, the fitted image, and corresponding 24,908-CP T-mesh of the LAST fitting. Bottom: fitting efficiency analysis against accuracies with a data scale of 512 ✕ 512 pixels (**a**), and analysis against image resolutions with a fitting RMSE of 0.022 (**b**). Dashed trend lines are fitted using moving averages.

We follow the convention of the state-of-the-art research [33] to fit the 8-bit RGB Lena image using the proposed algorithm. Figure 11 (top) presents typical fitted T-meshes of the two algorithms with an equivalent RMSE of 0.03. The results show that the LAST fitting produces a significantly simpler T-mesh than the split-connect fitting using only 80% control points of the latter. An efficiency analysis against fitting accuracy and sample set sizes was conducted. The results in Figure 11 (bottom) show that LAST is about twice to even over fourfold faster than the split-connect algorithm in high-accuracy scenarios.

## 5. Conclusions and Future Work

In this paper, a locally accelerated stitching T-spline, known as LAST, fitting algorithm is proposed for fast, large-scale freeform point cloud fitting. The method first segments a large-scale input point cloud into small local patches. Then it fits each patch of data individually to a T-spline through a locally accelerated fitting scheme. Finally, the individually obtained T-splines are stitched to a global output using a local optimization scheme around patch boundaries. The local fit-and-stitch strategy breaks the efficiency bottleneck of the existing global and local fitting algorithms about data scales. LAST also results in concise T-mesh structures with a considerable balance between fitting efficiency and accuracy control by preserving the local support characteristics of CPs.

The efficiency performance of LAST has been validated through experiments compared to a global, a local, and a split-connect T-spline fitting algorithm. The results show that the computational speed is about two0to-eightfold faster than the three selected latest algorithms. In the classical Lena RGB image study, LAST is shown to be at least two times faster than the split-connect algorithm, using fewer than 80% CPs of the latter. The proposed method can also be applied with advanced T-spline models, such as AST-splines, and hierarchical T-splines, providing a corresponding mesh refinement rule.

The solution to the issue of larger errors in high-gradient regions for LAST will be explored and discussed in depth in our future work. Simultaneously, considering the excellent characteristics of optical freeform surfaces such as feature sparsity and circular symmetry, NURBS has been widely utilized in their parameter representation. Therefore, integrating LAST into the fitting of optical freeform surfaces will be a challenging task for future research.

## Data Availability

Data are contained within the article.
